# Identifying and addressing methodological incongruence in phylogenomics: A review

**DOI:** 10.1111/eva.13565

**Published:** 2023-06-06

**Authors:** James F. Fleming, Alberto Valero‐Gracia, Torsten H. Struck

**Affiliations:** ^1^ Natural History Museum University of Oslo Oslo Norway

**Keywords:** branch length heterogeneity, compositional heterogeneity, incongruence, phylogenetic artefacts, phylogenetics, phyloinformatics, site saturation, software

## Abstract

The availability of phylogenetic data has greatly expanded in recent years. As a result, a new era in phylogenetic analysis is dawning—one in which the methods we use to analyse and assess our data are the bottleneck to producing valuable phylogenetic hypotheses, rather than the need to acquire more data. This makes the ability to accurately appraise and evaluate new methods of phylogenetic analysis and phylogenetic artefact identification more important than ever. Incongruence in phylogenetic reconstructions based on different datasets may be due to two major sources: biological and methodological. Biological sources comprise processes like horizontal gene transfer, hybridization and incomplete lineage sorting, while methodological ones contain falsely assigned data or violations of the assumptions of the underlying model. While the former provides interesting insights into the evolutionary history of the investigated groups, the latter should be avoided or minimized as best as possible. However, errors introduced by methodology must first be excluded or minimized to be able to conclude that biological sources are the cause. Fortunately, a variety of useful tools exist to help detect such misassignments and model violations and to apply ameliorating measurements. Still, the number of methods and their theoretical underpinning can be overwhelming and opaque. Here, we present a practical and comprehensive review of recent developments in techniques to detect artefacts arising from model violations and poorly assigned data. The advantages and disadvantages of the different methods to detect such misleading signals in phylogenetic reconstructions are also discussed. As there is no one‐size‐fits‐all solution, this review can serve as a guide in choosing the most appropriate detection methods depending on both the actual dataset and the computational power available to the researcher. Ultimately, this informed selection will have a positive impact on the broader field, allowing us to better understand the evolutionary history of the group of interest.

## 1 INTRODUCTION

**FIGURE 1:**
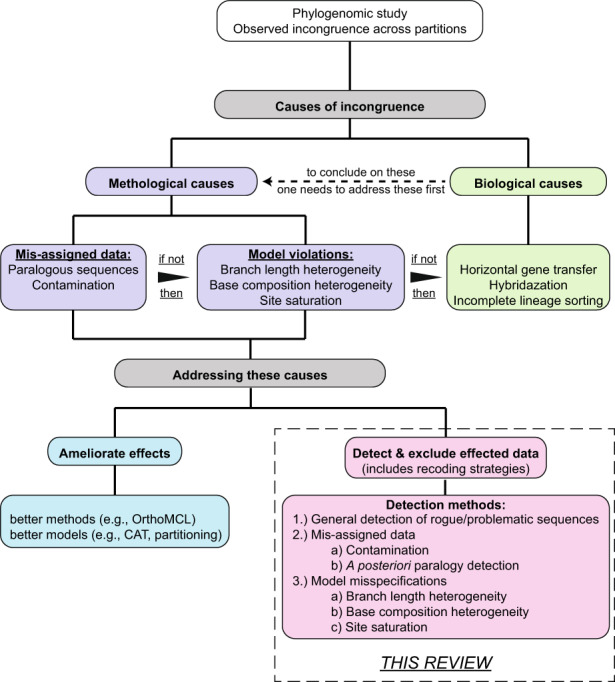
An introduction to the review, showcasing the many stages of phylogenetic analysis, and areas where potential incongruence might occur. This review is focused on methodological causes of incongruence.

Phylogenetics, the study of the relationships between groups of organisms, is a field that has seen a great increase in interest in the aftermath of the genomic revolution (Espinosa de los Monteros, 2020; Smith et al., 2020). One significant way in which the scientific community interprets the degrees of those relationships is through the bifurcating phylogenetic Tree of Life (Song & Hein, 2004). Given the vast amount of genomic and transcriptomic data that has recently become available for phylogenetic studies, it has been suggested that the goal of obtaining the most likely topology of the phylogenetic tree per se will soon be in our grasp (Gee, 2003). However, with increasing data and across many different studies, we now understand that simply procuring additional data will not answer many of the pertinent questions that remain about the history of life (Hillis et al., 2003; Philippe, Brinkmann, Lavrov, et al., 2011). Indeed, we appear to have entered the age of ‘true incongruence’, as proposed by Jeffroy et al. (Jeffroy et al., 2006), where our methods of analysis are more integral to our interpretations of hypotheses as opposed to simply adding more data. Especially in genomic research, while it can be important to include as much data as possible to obtain an accurate representation of all aspects of the evolutionary history of taxa, species or populations (Heath et al., 2008; Hillis et al., 2003; Zwickl & Hillis, 2002), in many situations, current methods of approximating evolution through time struggle with both implicit and explicit assumptions which can mislead the reconstruction of biological data. Such misleading effects due to the applied methodology should be minimized. Hence, we still need to consider the different causes of incongruence in this respect and assess whether they are due to biological causes or methodological shortcomings (Figure 1).

Roughly speaking, the biological causes of incongruence can be grouped into three distinct categories: horizontal gene transfer, hybridization, and incomplete lineage sorting. All three are population genetics level phenomena, but these can have great bearing even at the large‐scale macroevolutionary level (Mossel & Roch, 2010; Philippe & Douady, 2003; Rothfels, 2021). Horizontal gene transfer is the transfer of a small proportion of the genome of one species to that of another species in a horizontal manner: contemporaneously between individuals rather than vertically through time and descent. In hybridization, the genome of the offspring is the result of the successful reproduction of two previously reproductively isolated species. In the case of incomplete lineage sorting, the standing genetic variation of the ancestral species inherited by the immediate descendant species has not been sorted out prior to a speciation event, and so this genetic variation is passed on to these new reproductively isolated lineages. Eventually, the process of lineage sorting will be completed, but the distribution of the allelic variations of the affected genes across these species will not necessarily reflect their phylogenetic relationship, but rather the unique history of the variants themselves, even encompassing the random loss of forms of those variants (Maddison & Knowles, 2006). In any case, the history of individual genes and the history of the species, thereby, are not always the same. Especially, distinguishing incomplete lineage sorting from hybridization can be a difficult task, as interbreeding between two partially diverged descendant populations can create a similar phylogenetic signal to incomplete lineage sorting (Joly et al., 2009). A more detailed review of these phenomena and how they can be detected can be found elsewhere (Mirarab et al., 2014). However, before one can conclude biological causes, one must first ascertain whether the incongruence stems from methodological issues in the reconstruction of the trees (Figure 1). Only if such methodological causes can be excluded, biological conclusions can be safely drawn.

The methodological causes can be partitioned into two categories: misassigned data and model violations (Figure 1). Misassigned data are data which are assumed to fulfil the orthologycriterion (i.e. that they are related to each other due only to speciation events) but are, in reality, related in another fashion. In the case of paralogous sequences, these genes are grouped as orthologous sequences together, but are also related to one another by shared gene duplication events. Thereby, trees reconstructed from this data do not only reflect speciation, but also gene duplication events. While such reconstructed histories might be interesting for understanding the evolution of gene families, they are misleading when it comes to understanding the evolution of taxa, species, or populations if the goal is to obtain the most likely topology of the species tree. On the contrary, misassigned data due to contamination results from biological material that is not part of the intended sample being misidentified as representative of the target species and this should be generally avoided (Bourlat et al., 2003; Yoshida et al., 2017).

Incongruences caused by model violations, however, are the result of the empirical data violating the assumptions of the applied models of evolution utilized in phylogenetic reconstructions. As such, the phylogenetic reconstruction is misled and does not reflect the most likely topology of the phylogenetic tree per se. While any model to date (and probably always) is only an approximation and simplification of the complex process of evolution, model violations should nonetheless be kept to a minimal, acceptable level as best as possible.

**FIGURE 2:**
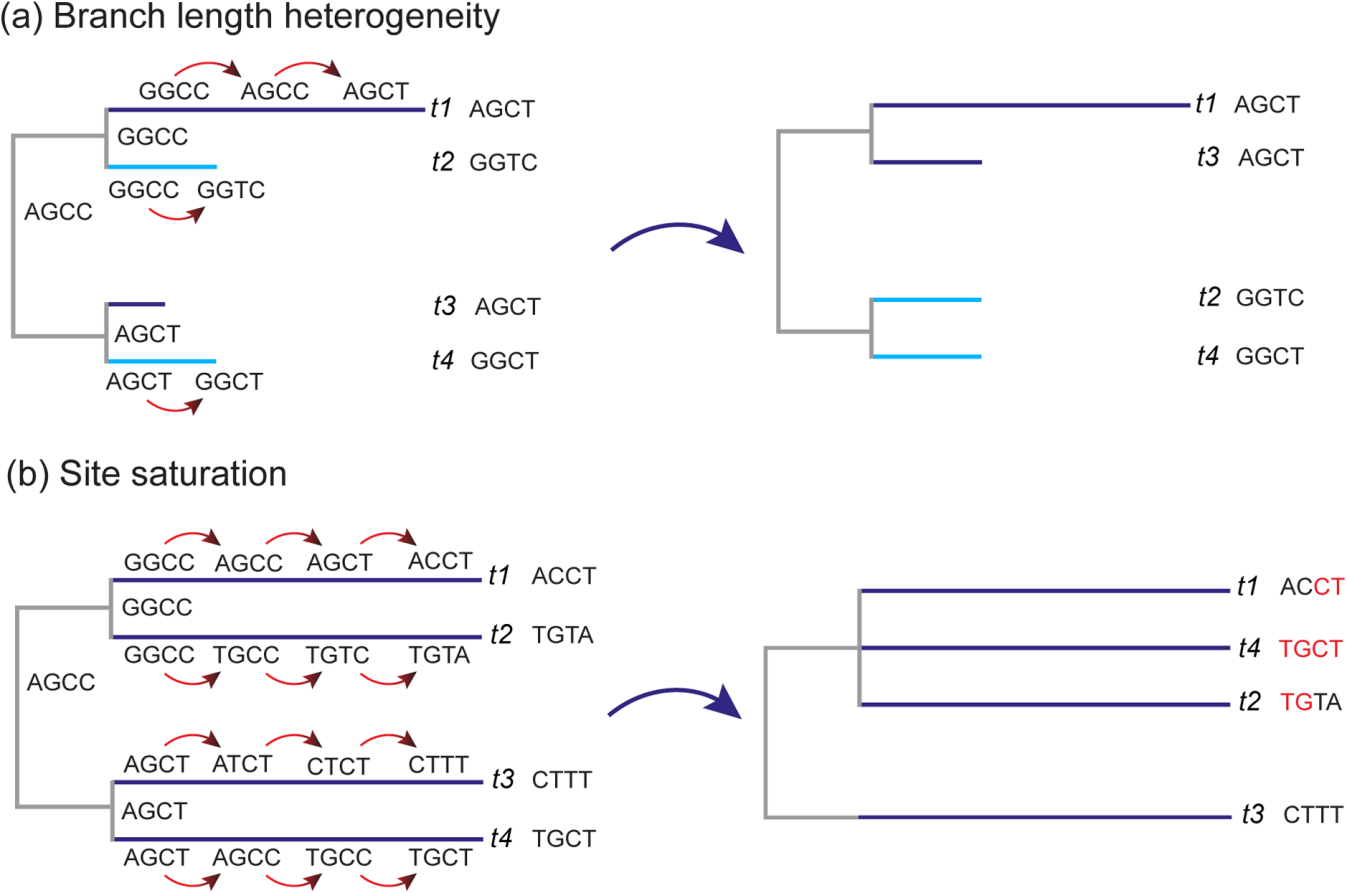
A figure depicting the key differences between branch length heterogeneity (Panel a) and site saturation (Panel b). The ‘true’ trees are depicted on the left, with the nucleotide changes along the branches, where the artefactual topologies are depicted on the right. In Panel a, multiple changes along the topmost branch *t1* result in a nucleotide combination that resembles the short branch *t3*, which is likely to cause the two branches to resolve as sister to one another in phylogenetic reconstruction. Meanwhile, under site saturation (Panel b), any signal in the data has been lost due to multiple large changes occurring along branches t1–t4 within the lineage. The artefactual signal (highlighted in red) favours a polytomy of *t1*, *t2* and *t4* together, to the exclusion of *t3*.

One well‐known model violation is branch length heterogeneity (also known as long branch attraction; Foster, 2004; Lyons‐Weiler & Takahashi, 1999; Nickrent et al., 2004; Philippe et al., 2005, 2007; Philippe, Brinkmann, Copley, et al., 2011; Redmond & McLysaght, 2021; Whelan et al., 2015). In this case, some taxa of the dataset have substantially longer branches in all or some of the partitions of the dataset relative to the other taxa present, and accordingly, the assumption of stationarity is violated. Stationarity means that the phylogenetic model assumes that the rates of change between nucleotides or amino acids are the same over the length of the tree (Jermiin et al., 2016). When the variation in evolutionary rate in a dataset is disproportionately located in a certain taxonomic clade this assumption can be violated. This could mean that a taxon in a dataset evolved much more rapidly than others, or that there is a great taxonomic distance between groups on the tree due to either a lack of sampling or an extinction event removing intermediary relatives. This can cause errors where these sequences cluster together in a single part of the tree, thus causing considerable distortions of the topology throughout the rest of the dataset (Figure 2). Long‐branch attraction is a significant problem as it leads to the inference of false, highly supported topologies (Felsenstein, 1978).

Another major cause of incongruence is the broad umbrella term of base composition heterogeneity. Phylogenetic models usually assume that the composition of sequences within the source dataset is broadly homogeneous (Whelan & Goldman, 2001). This means that they possess the same proportion of nucleotide bases or amino acids across the dataset, though these may occur at different site positions through time. Another additional assumption is reversibility, meaning that the process of nucleotide and amino acid change is undirected (i.e. that the probability of one nucleotide or amino acid exchanging with another is the same in both directions; Jermiin et al., 2016). In the case of base composition heterogeneity, when one or both assumptions are violated by some or all taxa, it can potentially cause topological and branch‐length reconstruction errors (Foster, 2004).

Finally, site saturation occurs when particular sites within a dataset are prone to change more frequently than assumed by the model (Lartillot et al., 2007). This can lead to the phylogenetic signal of those sites being obscured, or even lost. As a result, taxa are grouped together based on convergently evolved character states and branch lengths are substantially underestimated.

To deal with methodological issues, two procedures have historically been applied. One is to further improve our methods and develop more sophisticated modelling approaches to approximate the process of evolution (Figure 1). For example, for ameliorating model violations due to branch length or base composition heterogeneity, site‐specific models can be used (Foster, 2004; Lartillot et al., 2007). Model selection, including the use of partitioning schemes, prior to phylogenetic analyses is also useful in this regard (Kainer & Lanfear, 2015; Kelchner & Thomas, 2007; Posada & Buckley, 2004; Sullivan & Joyce, 2005). Model specification programs such as Modeltest‐NG (Darriba et al., 2020) and Modelfinder (Kalyaanamoorthy et al., 2017) have arisen to assist in this decision process to select the most optimal model of a set of models using information criteria based on the Akaike Information Criterion (AIC) or the Bayesian Information Criterion (BIC; Neath & Cavanaugh, 2011; Parzen et al., 1988; for a more detailed review of these aspects dealing with the methodological issues from a modelling perspective, see Kapli et al. [2021]).

However, these improved methodologies usually come at the cost of computational demands and despite the tremendous growth of available computational power, there is often a limit on the amount of data that can be included in a given analysis. Hence, data selection must be employed in one way or another. Moreover, this often entails determining many more parameters from a more restricted dataset. This can result in inconsistency as the methods try to maximize the amount of information accommodated by the model and hence are prone to overfitting data, which may potentially result in erroneous topologies (Huelsenbeck, 2004). Recently, even the concept of overfitting itself has become an important topic of analysis and conversation: some studies suggest that more modern profile mixture models, such as the C‐category models, may be robust to overfitting errors even on small datasets, and perform better than less complex models (Baños et al., 2022). Furthermore, while overfitting could be a concern in many current Bayesian phylogenetic software implementations, it is not necessarily a concern to Bayesian phylogenetic reconstruction itself, and careful selection of priors (such as the proportion of invariant sites, expected distribution of branch lengths and equilibrium base frequencies) might remove the issue entirely (Fabreti & Höhna, 2022).

Alternatively, it has been suggested to work on the data side of the problem. Given the vast amount of data available, one can select the data that best fit the assumptions of the models given the question at hand (Figure 1; Lemmon & Lemmon, 2013). The goal of this approach is to detect the data (taxa or partitions), which most strongly violate the model assumptions, and exclude these from the analyses (Fleming et al., 2020). In theory, excluding data that confounds the model will minimize the misleading signal due to reconstruction artefacts. This will ultimately also allow for the application of more sophisticated phylogenetic reconstruction methods while optimizing computational resources (Fleming et al., 2020; Heath et al., 2008; Jiang et al., 2014). Moreover, it allows us to explore how and why different misleading signals affect tree reconstruction. Topologies determined this way can then build the foundation of further analyses (Fleming et al., 2020; King & Lee, 2015; Litsios & Salamin, 2012; Misof et al., 2013). It should be mentioned here that the exclusion of data does not only mean the exclusion of taxa or partitions, but also recoding strategies. Recoding strategies compress data states down to a smaller number to reduce the effects of base composition heterogeneity—for example, treating the 20 amino acid states as 6 states based on the amino acid's physical properties, also known as Dayhoff‐6 recoding, is one of many available recoding strategies.

To appropriately detect and exclude potentially misleading artefacts, several methodologies have been developed (Aberer et al., 2011; Fleming et al., 2020; Mirarab et al., 2014; Wilkinson, 2006; Zhong et al., 2011). While the methods and effects of the biological causes and ameliorating strategies have been comprehensively reviewed recently (Kapli et al., 2021; Mirarab et al., 2021), a modern review is lacking that reflects on recent developments of the methods and approaches that can be employed to detect potentially misleading data and signals (Lemmon & Lemmon, 2013). In addition, phylogenetics has in general, but especially with respect to detection methods, a large glossary of key terms to codify specific problems and a great deal of often complex statistical and probabilistic mathematics. Understandably, this background of technical jargon can sometimes seem convoluted to those who are not already highly invested in the field (Lozano‐Fernandez, 2022).

The intent of this review is to present an overview of currently available programs to detect different misleading aspects in a phylogenomic dataset (dashed box in Figure 1), their advantages and disadvantages and the progress in the field since the Lemmon & Lemmon review (Lemmon & Lemmon, 2013). Furthermore, this review will provide a primer for those unfamiliar with such phylogenomic detection methods. To do so, we take a practical, problem‐first approach, with each section addressing a different form of incongruence, explaining the problem in clear language, and then offering software and methodological suggestions to tackle the discussed issue, whilst explaining the criteria and approaches that these methods take to achieve their goal. This review hopes to build on other recent, comprehensive overviews of key aspects of the field by Mirarab et al., Kapli et al. and Lozano‐Fernandez (Kapli et al., 2021; Lozano‐Fernandez, 2022; Mirarab et al., 2021), providing a solid and exciting introductory foundation to the often‐confusing world of phylogenetic analysis.

## 2 GENERAL DETECTION OF ROGUE AND PROBLEMATIC SEQUENCES

Rogue and problematic sequences are two different sequence subsets here grouped together due to their similar appearances within datasets and their similar classification—that is, as sequences that can be identified as responsible for errors in datasets (Aberer et al., 2011, 2013; Fleming et al., 2020). However, the way in which these errors manifest is notably different. A rogue sequence is a sequence which cannot be robustly placed anywhere within a phylogenetic topology due to either ambiguous or insufficient phylogenetic information in the dataset, and as such is prone to moving around the tree (Aberer et al., 2013; Wilkinson, 1996). Meanwhile, a problematic sequence is one that cannot be accommodated given the current model and dataset, and thus distorts the effect on the resultant tree, moving other sequences towards or away from it (Fleming et al., 2020). One aspect to note here is that whilst there are a variety of ways to detect such sequences, these methods do not tell us the reason these sequences disrupt the phylogeny the way that they do. To understand why we must investigate the cause of incongruence (Figure 1).

### 2.1 Bootstrapping, stability and rogue sequences

Rogue sequences are best identified by using bootstrapping methods (Aberer et al., 2013). Bootstrapping is a way to test the robusticity of a phylogenetic hypothesis by determining whether the same bipartitions occur through the use of a set of ‘pseudoreplicates’—new datasets based on the original that remove certain sites and multiply others to produce a dataset of the same size as the generating dataset (Hillis & Bull, 1993). If some sites in the dataset are biased towards a particular topology, the bootstrap method should be able to report said bias. Bipartitions that do not robustly occur throughout the pseudoreplicate bootstrap set are not supported by a majority of sites and so will not be recovered with a high bootstrap value, thus potentially identifying problems within the original dataset. Bootstrapping measures robusticity, and not accuracy, which is worth considering when evaluating phylogenetic topologies—a sequence may be reliably recovered across the pseudoreplicates in an incorrect place. This incorrect placement may be due to low‐quality data, poor modelling, or insufficient information given to the model, but the node will still possess a high bootstrap value (Hillis & Bull, 1993). Therefore, a high bootstrap value at a given node is a measure of how consistently this bipartition is resolved, not necessarily a measure of whether that bipartition is ‘true’.

A more thorough and sophisticated exploration of bootstrap values is utilized by the PABA (Partition Addition Bootstrap Alteration) approach (Struck, 2007, 2014; Struck et al., 2006) which uses a host of statistical tests to explore and explain the bootstrap value distribution across a dataset. In this approach, the change in bootstrap values at a given node is assessed in comparison to the addition of a new partition to an existing dataset. This is done systematically for each possible node, each partition in the dataset, each possible order of addition and each possible composition of the dataset the partition is added to. This allows us to explore whether the bootstrap support at each node increases or decreases when a partition is added and whether the confounding signal of that partition can be overcome with more data or is persistent across the dataset. This also reveals problematic nodes, and hence possible rogue sequences in partitions, but can also be applied to other measures of nodal support such as Bremer support or posterior probabilities.

Following this, PABA can then apply further statistical tests to assess the support for the conclusions of a phylogenetic dataset (Struck, 2007; Struck et al., 2006). A Wilcoxon‐Signed‐Rank test (Lee, 2000; Lee & Hugall, 2003; Macey et al., 1999; Templeton, 1983; Whitlock & Baum, 1999) can be used to assess whether the positive contribution of a partition outweighs, if present, its negative impact on a given set of nodes, which can allow for the detection of problematic partitions. The results of this test can also guide the decision of whether an entire partition should be excluded from the analysis instead of just a few rogue sequences from such a partition. Meanwhile, the permutation test (Farris et al., 1995; Thornton & Desalle, 2000) assesses the significance of the results of each partition at each node and orders of addition. For this permutation test, positions are randomly assigned to partitions of the same sizes as the predefined partitions used for the calculation of the original values. Then the same analyses are conducted as for the original partitions. Thus, the test can reveal if the value found for a partition at a node and order of addition can be obtained just by chance by a randomly partitioned version of the same data set. While this approach is very useful for small datasets with a limited number of partitions (Bruyndonckx et al., 2009; Regier et al., 2008; Sung et al., 2007), it is too computationally demanding for large phylogenomic datasets. For example, a dataset with 100 taxa, 100 partitions and 100 pseudoreplicates requires more than 10^34^ tree searches (Struck, 2007).

Two faster and more computationally efficient methods than PABA that also rely on bootstrapping procedures to identify rogue sequences are Roguenarok (Aberer et al., 2011), and the leaf stability index (Thorley & Page, 2000; Thorley & Wilkinson, 1999). Roguenarok takes as input a file of bootstrap trees. It then uses these trees to calculate the RBIC—the Relative Bipartition Information Criterion—of the phylogeny, which it defines as the sum of all support values divided by the maximum possible support over the set of taxa. As such, the exclusion of rogue taxa should result in a notable increase in the RBIC, allowing Roguenarok to quantify the change in robusticity of the dataset that can be produced by the exclusion of a given bipartition. Roguenarok then attempts to determine the minimal number of taxa that can be removed from the bipartition to produce the largest increase in the RBIC. As bipartitions may occur on internal branches, using the RBIC value too bluntly may result in overpruning a dataset, as some taxa may contribute much more highly to decreases in RBIC than others. Subsequently, Roguenarok outputs a list of taxa alongside the RBIC increase that will likely result from the removal of the taxa. Taxa with large values here are considered prime candidates for pruning in future analyses.

**FIGURE 3:**
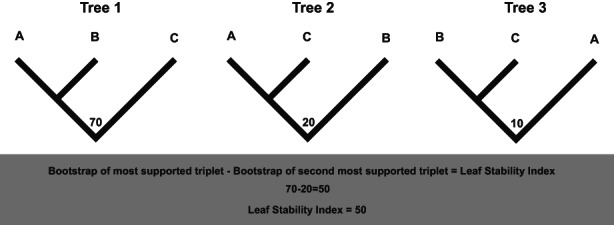
A figure describing the Leaf Stability Index. By subtracting the bootstrap values of the two most highly supported bootstrap topologies of a triplet (which has three possible topologies) from one another, we can determine the stability of a leaf. A B and C represent a rooted triplet tree, and so they may be taxa, or a selection of sequences collapsed together.

The leaf stability index (Thorley & Page, 2000; Thorley & Wilkinson, 1999) is implemented in Roguenarok and PhyUtility (Aberer et al., 2011; Smith & Dunn, 2008). For each taxon individually, each possible triplet stability is calculated and the average of these is the leaf stability for the taxon. Triplet stability is the difference in bootstrap support between the two strongest supported topologies of the possible rooted 3‐taxon statements for each triplet, as any phylogenetic problem can be simplified to a 3‐taxon statement by collapsing groups (Figure 3). Hence, while both measurements use bootstrap as their basis, the calculations are slightly different. However, both methods come with significant disadvantages. Roguenarok's focus on pruning based on tree support to improve a bootstrap score (Felsenstein, 1978; Goloboff & Szumik, 2015) can result in erroneous, but well‐supported topologies being favoured by the algorithm, thus removing the uncertainty that would normally be expressed by a low bootstrap score and creating a dataset with higher local likelihood optima. Meanwhile, the leaf stability index (Smith & Dunn, 2008) is much slower and computationally intensive.

Recently, an alternative to the traditional way of calculating bootstrap values has emerged, the transfer bootstrap (Lemoine et al., 2018). This new value can be assessed by all the bootstrap metrics discussed above and is far more resistant to disruption by rogue sequences. It achieves this by considering the degree of difference between taxa placement in the bootstrap trees across the dataset, known as the transfer index, rather than merely counting whether a given bipartition is present. This means that a sequence that moves actively across a bootstrap dataset—a rogue sequence—will appear with low transfer bootstrap support without necessarily decreasing the support of surrounding, more stable, nodes. The transfer bootstrap is implemented in booster (Lemoine et al., 2018), which additionally has an online platform for ease of use (Table 1) that accepts the bootstrap support files generated by RaxML, IQ‐Tree and most other phylogenetic software.

**TABLE 1:** An overview of the software and methods presented throughout the review, divided by the methodological incongruence they attempt to address.

Name	Software or method?	Citation
Model selection		
Modeltest‐NG	Software	Darriba et al. (2020)
ModelFinder	Software	Kalyaanamoorthy et al. (2017)
Akaike information criterion	Method	Parzen et al. (1988)
Bayesian information criterion	Method	Neath and Cavanaugh (2011)
Rogue sequence		
RoguenaRok	Software	Aberer et al. (2011)
Phyutility	Software	Smith and Dunn (2008)
Booster	Software	Lemoine et al. (2018)
Bootstrapping	Method	Hillis and Bull (1993)
Partition addition bootstrap approach	Method	Struck et al. (2006)
Permutation test	Method	Thornton and Desalle (2000)
Leaf stability index	Method	Thorley and Wilkinson (1999)
Transfer bootstrap	Method	Lemoine et al. (2018)
Problematic sequences		
Canary sequence method	Method	Fleming et al. (2020)
Partition Coalescence support	Method	Chandonia et al. (2004) and Liu et al. (2010)
Contamination		
Blobtools	Software	Laetsch and Blaxter (2017)
CroCo	Software	Simion et al. (2018)
Paralogy		
OrthoMCL	Software	Li et al. (2003)
PhylotreePruner	Software	Kocot et al. (2013)
TreSpeX	Software	Struck (2014)
Branch length heterogeneity		
K‐score	Software	Soria‐Carrasco et al. (2007)
Treeshrink	Software	Mai and Mirarab (2018)
Aligroove	Software	Kück et al. (2014)
Slow‐fast method	Method	Brinkmann and Philippe (2008)
Long branch score	Method	Struck (2014)
Locus‐specific sequence subsampling	Method	Rivera‐Rivera and Montoya‐Burgos (2016, 2019)
Compositional heterogeneity and site saturation		
Phylobayes posterior predictive tests	Software	Lartillot et al. (2009)
BaCoCa	Software	Kück and Struck (2014)
SRH test	Method	Naser‐Khdour et al. (2019)
Chi‐squared test for compositional heterogeneity	Method	Cummings (2004)
RCFV	Method	Kück and Struck (2014)
Patristic distance linear regression	Method	Nosenko et al. (2013)
Convergence values	Method	Kück and Struck (2014)
Saturation index	Method	Duchêne et al. (2021)
Recoding strategies	Method	Hernandez and Ryan (2019) and Smith et al. (2009)

### 2.2 Problematic sequences and support

Problematic sequences are ‘sequences that lack sufficient phylogenetic signal to be robustly resolved under the considered substitution model’ (Fleming et al., 2020). This means that they are likely to have a distorting effect and may well be robustly resolved despite being misleading. The presence of a large amount of phylogenetic noise in a dataset may result in topologies that appear novel but are in fact due to misleading contributions from a single sequence or a subset of sequences.

The canary sequence methodology (Fleming et al., 2020) attempts to identify and exclude these problematic sequences through a multi‐staged process of elimination. It works by the researcher a priori identifying a set of potentially problematic ‘sequences of interest’. These are either new sequences that the researcher wishes to introduce to an existing dataset to better understand their phylogenetic affinities or a particular group that in the literature has previously been identified as controversial (resolving in multiple positions within the tree between different analyses). Following this, a ‘base dataset’ consisting of previously published closely related sequences is compiled. The method proceeds by testing these sequences of interest by generating: (i) a single tree, which contains the entire base dataset and all of the sequences of interest (i.e. the ‘full tree’), (ii) a tree consisting of the base dataset and that sequence of interest (i.e. the ‘checking tree’). Sequences of interest that result in different positions between the full tree and their checking tree without altering the topology of the surrounding tree between those two conditions are identified as ‘canary sequences’. Canary sequences, thereby, are sequences that move in the presence of problematic sequences. Following this, (iii), a tree is constructed containing all the canary sequences, known as a ‘canary tree’. The canary tree is used as the basis for another round to test the remaining sequences of interest. Finally (iv), ‘canary checking trees’ are produced by constructing trees that use all the sequences present in the new canary tree plus a remaining sequence of interest. If a canary checking tree has a different topology than the canary tree, that sequence of interest is then excluded as problematic. This is because sequences that move the canary sequences are, as a single sequence, contributing a large amount of noise to the dataset.

**FIGURE 4:**
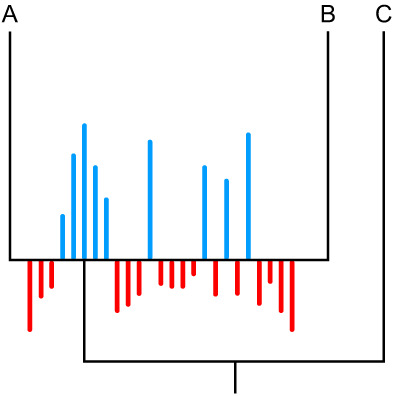
A figure depicting PCS, an optimality criterion method. Each partition's support for the bipartition presented at the node is presented as a magnitude. Support for the bipartition presented is in blue and support against is in red. The length of the bars represents the strength of the support in favour or against the bipartition. This strength can be measured in log‐likelihood units, compatible quartets or other support metrics. In this worked example, though the majority of partitions do not support the topology, the partitions that do support the topology are more confident in that support. This might warrant further examination of the dataset to assess whether that confidence is artefactual.

A faster method to assess support for large datasets and identify problematic sequences is to use gene trees and coalescence. Methods such as ASTRAL or MP‐EST use a metric known as the partitioned coalescence support (PCS), an optimality criterion (Chandonia et al., 2004; Gatesy et al., 2016; Liu et al., 2010). Like PABA, it summarizes the distribution of support across the partitions for a node, but the approach is noticeably different. For each node, there are two possible alternative resolutions in an unrooted tree (Figure 4). The PCS value of a node is equal to the number of gene trees (derived from partitions) that support that node vs. the number of gene trees that support an explicit alternative (that is to say, disregarding trees that support neither explicit hypothesis). Positive values indicate that the dataset is in support of the node, while negative ones indicate incongruence. The higher the value, the stronger the magnitude of support or incongruence. These values can then be explored as within PABA, but it does not allow assessment for interaction between partitions, as not all possible combinations of partitions are explored. On the contrary, this restriction to individual partitions, and an aversion to bootstrapping procedures, make it a very fast approach to establish incongruence across genome‐scale data (Gatesy et al., 2019). Significance tests have not yet been implemented for PCS.

ALE is another coalescent‐based method that attempts to reconcile the gene and species trees (Szöllősi et al., 2013). ALE takes as input a sample of gene trees and a rooted species tree. The input gene trees are then reconciled with the species tree—subjected to a likelihood estimation to determine whether the observed duplication, transfer and loss events (DTL events) are real or artefactual. In many cases, ALE reconciles a smaller number of these DTL events than interpreting the gene tree data alone would imply, suggesting that, with the lower amount of data available in single sequence alignments, misleading topologies are more likely to be returned (Szöllősi et al., 2013). However, ALE requires a confident species tree for its gene tree reconciliation to be equivalently confident, and to be able to accurately assess the true discord between the gene and species trees.

SpeciesRAX, meanwhile, attempts to use the gene tree to generate the species tree, considering duplication, gene transfer and loss (Morel et al., 2022). SpeciesRAX takes as input either a set of multiple sequence alignments or a set of gene trees. In the former case, it will generate a series of gene trees in RaxML‐NG (Kozlov et al., 2019) before embarking on its unique analysis. SpeciesRAX starts from a random rooted species tree and, taking into account the gene trees, attempts to optimize for a most likely species tree considering all observed gene tree inputs. It does this by first attempting to identify paralogous genes that exist within the gene trees, pruning them, and then using these simpler trees to identify an initial unrooted species tree. This tree is then rooted and rerooted in a likelihood search to obtain an optimized species tree. SpeciesRAX is fast, accurate and appears to be robust to paralogy within its gene trees, however, the paralogy pruning method does leave it open to incorrectly interpreting horizontal gene transfer as paralogy, which may be relevant for microbial datasets (Morel et al., 2022).

It is worth noting that exclusion‐based methods such as PABA, PCS, Roguenarok, PhyUtility, and the canary sequence approach (Aberer et al., 2011; Chandonia et al., 2004; Fleming et al., 2020; Smith & Dunn, 2008; Struck, 2014), whether assessing stability or support, work by removing data (taxa and partitions) from the dataset. This will result in more robust, confident, and well‐resolved topologies. However, the topologies may still be erroneous due to a lack of data or poor model specifications. These identification methods may be either positively misleading or negatively misleading. Positively misleading results will propose new phylogenetic hypotheses influenced by the effect of the method upon the source dataset, whereas negatively misleading results will resemble those generated from the source dataset (Fleming et al., 2020). Unfortunately, for sequences that cannot be accommodated by our current modelling, the negatively misleading results provided by robusticity measures that remove informative sequences through overzealous pruning may be more informative than the positively misleading results provided by including rogue sequences. When the methods presented here produce inaccuracies, they are more likely to produce an inaccurate but previously recovered topology rather than presenting a radically new, but still incorrect, hypothesis. In this respect, coalescence‐based methods such as ASTRAL, SpeciesRAX and ALE (Chandonia et al., 2004; Morel et al., 2022; Szöllősi et al., 2013) that allow researchers to explore conflicting hypotheses presented by their dataset more explicitly may present a more exciting future for detecting problematic sequences and discerning biological incongruence.

## 3 MISASSIGNED DATA

### 3.1 Contamination

Before beginning an analysis, the first step should be to ensure that the dataset itself is as free from contamination and as reliable as possible. This kind of dataset refining can take many forms depending on the intent of the analysis. Contamination is a major concern within phylogenetic analyses, as incorrectly identified sequences can cause numerous problems with phylogenetic inference and genomic predictions (Arakawa, 2016).

Thankfully, a number of tools exist to address this problem. BLAST, and especially its web application at NCBI (Altschul, 2001; Camacho et al., 2009), is perhaps the most widely used tool for contamination identification and plays a role in many of the other tools expanded upon later in this review. BLAST identifies short matches (‘words’) between the target sequence and a database and ranks the similarity between the target sequence and sequences in the database using an e‐value. This results in the most similar sequences having the e‐value closest to 0, where 0 is a perfect match. Many prebuilt databases using a vast amount of publicly available molecular data are available on multiple websites (e.g. EMBL and NCBI).

In these cases, each sequence is usually used blasted against a tailored dataset, which contains sequences of possible contaminants and the target taxon (Kocot et al., 2016, 2020; Laumer et al., 2015; Simion et al., 2018; Struck et al., 2014). The sequence will only be kept in the dataset and not pruned if the best hits match the target taxon and not a possible contaminant. Prior knowledge of possible contaminant sources such as endosymbionts, parasites, and hosts has been used to design these datasets. Alternatively, barcoding markers like 18S or COI have been used to detect possible contaminants first and then build the databases based on this information. However, these approaches depend either on prior knowledge and/or availability of data for the detection and screening procedures. Moreover, lack of sufficient data within a group might make the granularity of such a screening relatively coarse, for example, only at the phylum level instead of the genus or species level. This might be especially problematic when the contamination stems from cross‐contamination between closely related taxa during the sample preparation and sequencing process (Simion et al., 2018). Often closely related samples are processed together and if the differentiation between taxa is only possible at a higher taxonomic level, these contaminations cannot be detected. This might be especially problematic as these contamination sources are liable to be included in the same phylogenetic analyses as one another and might thereby lead to misleading conclusions that appear reasonable.

Blobtools (Laetsch & Blaxter, 2017) is a more comprehensive visualisation tool including contamination detection. To this end, the first step is to BLAST the entire target genome or transcriptome of a taxon against a large database such as the entire one from NCBI, which is not tailored towards specific taxa. The obtained hits from this comprehensive search are used to visualize which percentage of the dataset is likely to belong to your target taxon and how much is likely contaminated. Moreover, it allows data exploration at different taxonomic levels instead of the standard procedures, which are limited by the composition of the initial screening database. However, due to the search taking place against a much larger database, it is also much more computationally demanding. In addition to contamination screening, it also provides utility to measure coverage and annotate genome assemblies based on the results of the screening, making it a useful tool during these early, preparative stages of genome analysis. Finally, it can efficiently filter these contaminated sequences from the dataset based on either their taxonomic identification in the blast search, GC content and/or coverage (Challis et al., 2020).

To identify cross‐contamination, different methods might need to be employed. CroCo (Simion et al., 2018) is specifically designed to address this problem. It takes two inputs as sequence read data, and first uses BLASTn to establish a list of plausible contaminants and then assesses each suspicious sequence's expression level across the entire read file. Reads less highly expressed in one input compared to the other are considered contaminated (with the lower read expression being due to contamination of the data). This is most effective at middling levels of gene conservation—CroCo was found to have a 100% success rate in cases where the true orthologs showed more than 2% divergence, but in some hyper‐conserved sequences, CroCo may remove real reads as contamination (Simion et al., 2018). Similarly, CroCo struggles to distinguish between taxa that are very closely related, which can make it inappropriate to use in some cases of suspected cross‐contamination. In addition, most instances of cross‐contamination occur at sequencing facilities, and so for an end‐user researcher, the necessary comparative read files may not be available to provide CroCo with input.

### 3.2 A posteriori paralog detection

More insidious than contamination is paralog misidentification—here the sequence belongs to the organism, but is not the ortholog of that gene within the organism (Fleming et al., 2020). This can result in erroneous phylogenetic inferences for the same reason—the history of the gene is not the same as that of the label it has been assigned to.

Also based on BLAST, OrthoMCL (Li et al., 2003) is an orthology identification software that uses repeated, reciprocal BLAST searches to steadily identify group similarities between two or more species. These reciprocal BLAST searches are then clustered using a Markov Cluster algorithm to match those that are most similar to one another into sections of a likelihood space, each representing an orthologous group. This can make it particularly useful for defining new orthologs, or to find orthologs in groups where there is poor sampling that the study is supplementing. Some orthology identification tools, such as Orthofinder (Emms & Kelly, 2019) extend this principle—given an input dataset, Orthofinder will automatically infer rooted gene trees from the results of a search using either BLAST or DIAMOND—a method similar to BLAST that is much faster, in exchange for a minor loss in sensitivity, and so is preferred on huge metagenomic datasets (Buchfink et al., 2021). By using phylogenetic trees, Orthofinder allows users to immediately and easily identify potentially paralogous sequences in a phylogenetic context, and its sensitivity means that it additionally proves to be slightly more accurate when identifying orthologous groups (Emms & Kelly, 2019).

Prior to embarking on a large multi‐gene analysis, the analysis of single‐gene datasets to easily identify contamination and erroneously inferred orthologs is advised. Both PhylotreePruner (Kocot et al., 2013) and TreSpeX (Struck, 2014) use phylogenetic information derived from single‐gene phylogenies to identify issues with contamination, ortholog identification, and long‐branch attraction before moving to a multi‐gene phylogenetic analysis. PhylotreePruner first collapses nodes below a user‐designated support value to polytomies and then removes sequences from taxa that do not form a monophyletic group (indicating independent duplication in that taxa) if multiple sequences from the same taxa are present. This is a conservative approach to a clear phylogeny‐informed multi‐gene dataset but remains susceptible to removing true orthologs that are incorrectly resolved in the initial single‐gene tree due to the issues here discussed. However, it is particularly useful when used to trim a large dataset with potentially numerous misspecified orthologies.

TreSpEx (Struck, 2014), meanwhile, takes a similar approach, using single‐gene phylogenies to remove paralogs, but instead employs BLAST to detect them. If the two partitions of a strongly supported bipartition in a given single‐gene phylogeny do not correspond to the same best hit in a given BLAST search, this bipartition is flagged as potentially containing paralogs, and those sequences that do not correspond to the majority best hit are pruned from the dataset. Support for a bipartition is assessed by bootstrap thresholds defined by the user. Additionally, TreSpEx allows applying additional criteria for screening and pruning such as extremely long branches leading to the node in question or extremely short branches leading to the terminal taxa. The former can also be an indication of paralogy due to the duplication event having occurred long before the speciation event leading to this node. The latter is an indication of possible cross‐contamination as the sequences of two different species will be almost identical.

Both approaches in PhylotreePruner and TreSpEx cannot detect problems that arise due to single terminal taxa as they rely on bootstrap support for this initial detection. Approaches using distance‐based measurements can accomplish this (Misof et al., 2014). However, this can also result in the pruning of taxa that are not affected by paralogy, but just exhibit longer branches and have increased substitution rates. In such approaches, the distance of each taxon to a set of reference taxa is determined and compared to the distances the reference taxa have among each other. If the distance is larger than an a priori fixed threshold, the taxon is excluded from the respective dataset.

The alternative approach, when orthology inference is too difficult, is to rely on an approach that is robust to paralogy, which has recently been robustly reviewed elsewhere (Smith & Hahn, 2021). These methods have a long history (Slowinski et al., 1997) but have struggled to reach popular usage due to the desire to use orthologous genes for gene tree analyses specifically, and the lack of whole‐genome scale data (Smith & Hahn, 2021). However, in an era of high‐quality genome sampling, and alongside more sophisticated methods (Rabiee et al., 2019; Smith et al., 2022), they may play an important role in species inference in the future.

## 4 MODEL MISSPECIFICATIONS

### 4.1 Branch length heterogeneity and long branch attraction

Branch length heterogeneity, also known as long‐branch attraction (Figure 2), is not caused by the existence of long branches themselves, but rather by the difference in their length relative to the rest of the dataset (Lyons‐Weiler & Takahashi, 1999; Struck et al., 2014). Branch length is an indication of the distance between nodes and leaves of the tree. A long branch indicates a great amount of change from the shared ancestral node. Where this change is large compared to other branches in the dataset, this can obfuscate the true topology of the tree (Figure 2), as the independent change along the long branch can make it appear artefactually similar to other groups in the dataset.

Of all potential causes of incongruence discussed in this review, branch length heterogeneity is probably the most controversial and most heavily discussed (Foster, 2004; Lyons‐Weiler & Takahashi, 1999; Nickrent et al., 2004; Philippe et al., 2005, 2007; Philippe, Brinkmann, Copley, et al., 2011; Pisani et al., 2015; Redmond & McLysaght, 2021; Susko & Roger, 2021; Whelan et al., 2015). Even prior to the common usage of Maximum Likelihood and Bayesian statistical techniques in phylogenetics, discussion around ways to identify and counter long‐branch attraction and quantify the ‘Felsenstein Zone’—the probabilistic area where two taxa with long branches artefactually gravitate towards one another—was already commonplace (Bergsten, 2005; Felsenstein, 1978; Huelsenbeck, 1997; Philippe et al., 2005). As branch length is optimized concurrently with the topology (i.e. the partition of relationships), even correctly identifying long branches can be problematic (Kennedy et al., 2005; Lyons‐Weiler & Hoelzer, 1997).

The most common way to deal with long‐branch attraction in a dataset is through the addition of more taxa. The addition of taxa that are closely related to the long‐branched taxa will ‘break’ the long branches by reducing them in length (Zwickl & Hillis, 2002). However, this is not always possible—depauperate groups, for example, possess long branches separating them from their closest relations because the taxa that would break down those branches have gone extinct and so can no longer be sampled. In addition, even if the researcher is aware of the presence of long‐branch attraction in their dataset, appropriately targeting, collecting, and sequencing further sampling may be logistically difficult. Furthermore, long branches can be cryptic, especially in under‐sampled data. Despite their name, long branches may not appear uncharacteristically long in the, artefactual position they are recovered in the topology (Fleming et al., 2020; Ramirez et al., 2016; Schnitzler et al., 2012). As such, the presence of branch length heterogeneity in the real data is not necessarily obvious, so targeted taxon sampling may not be possible.

Methodologically identifying long branches can be problematic and highly subjective (Kennedy et al., 2005; Lyons‐Weiler & Hoelzer, 1997). One approach which is still applied to counter the problem of long‐branch attraction is the exclusion of the fastest evolving genes—also known as the slow‐fast method (Brinkmann & Philippe, 2008). This approach implies that the evolutionary rate of the entire gene is a good predictor of branch length heterogeneity, as the evolutionary rate is increased in the affected species. However, the evolutionary rate can also increase across all taxa in a dataset, which would not lead to branch length heterogeneity and accordingly, the evolutionary rate is not necessarily a good predictor (Bergsten, 2005; Kocot et al., 2016; Kück et al., 2012; Struck et al., 2014). Fortunately, approaches that are more objective have been developed in assisting the identification of long branches and branch length heterogeneity in the dataset independent of whether the long branches are caused by high evolutionary rates, sampling or depauperate groups.

The K‐Score (Soria‐Carrasco et al., 2007) is a useful measure of branch length heterogeneity in a dataset. The K‐Score compares two given trees against one another by creating a scaled ratio of their total branch length distance. This means that similar trees differentiated only by an abnormally large increase in branch lengths will become very clearly noticeable to the K‐Score metric. As such, it is a useful tool when comparing new hypotheses and when adding new data to a previously established dataset to assess the relative change in branch lengths across the topology. Still, the K‐Score first needs two trees to compare against one another and, in the absence of a clear prior null and alternative hypothesis, will be unable to identify the exact location of the artefact within the dataset itself.

Another approach is the long branch (LB) score implemented in TreSpEx (Struck, 2014), which is essentially a further development of the tip‐to‐root distance that has been applied before to detect taxa with a long distance to the root (Rodríguez‐Ezpeleta et al., 2007). This allows us to determine taxa, trees and datasets which are affected by branch length heterogeneity (Kocot et al., 2016; Struck et al., 2014). The LB score is essentially the deviation of the average pairwise distance of a taxon to all other taxa across all pairwise distances. The higher the LB score, the stronger the deviation of the taxon from the mean and the longer the branches leading to the taxon. As the score is distance‐based and normalized on the mean, it can be compared across trees independent of their topology, number of taxa, and overall rate of evolution (Struck, 2014). Moreover, in contrast to the tip‐to‐root distances, it is independent of the root of the tree. LB scores were originally proposed for tree‐based related distances, but they can be applied to any distance matrix as well. The LB score itself is a taxon‐specific measurement and as such, one can use it to detect long‐branched taxa or sequences in a dataset. Additionally, measurements for trees, genes or partitions can be derived by calculating either the mean value of the upper quartile or the standard deviation of the relevant LB scores. Both measurements are highly correlated (Struck et al., 2014) and the higher they are, the more branch length heterogeneity is present in the entire tree. Thus, a comparison across entire trees is possible (Kocot et al., 2016).

The *k*‐shrink optimization problem implemented in TreeShrink (Mai & Mirarab, 2018) is principally similar to the LB score. It determines the sequence pairs in the tree which possess the maximum distance between any two leaves. This is also known as the tree diameter and, like the LB score, it is independent of the root. It then iteratively checks if excluding one of the sequences from these diameter‐length pairs significantly reduces the tree diameter. This allows it to calculate a ratio of the tree diameter with respect to the excluded sequence. To be able to detect long internal long branches and not only long terminal branches, *k*‐shrink further iterate this procedure over a user‐defined number of sequences removing them each time. This should result in a significant decrease in tree diameter that is consistent across iterations if long internal branches are removed. The sequences detected this way can be excluded from the analysis. In contrast to the LB score, gene‐specific scores cannot be calculated using this approach.

Another method to detect long‐branched sequences in a gene or partition has been implemented in the LS^3^ (Locus Specific Sequence Subsampling) and LS^4^ approaches of LS^X^ (Rivera‐Rivera & Montoya‐Burgos, 2016, 2019). The first implementation of this approach was LS^3^ (Rivera‐Rivera & Montoya‐Burgos, 2016). The general goal of this approach is to detect a subset of sequences exhibiting homogeneous evolutionary rates and then to flag all others as potentially possessing problematically high or slow evolutionary rates. Hence, it addresses branch length heterogeneity from both sides. The procedure is based on user‐provided input trees, which pre‐define lineages of interest with polytomies between both them and an outgroup, and likelihood ratio tests (LRT; Anisimova & Gascuel, 2006). The branches of the input tree are optimized and then LRT compares a model assuming a homogeneous evolutionary rate among all ingroup lineages with a model that allows for independent rates for each ingroup lineage. If the homogeneous model is rejected, the sequence with the longest distance from the root of the polytomy to the tip is removed and the input tree is adjusted accordingly. This procedure is repeated iteratively until the homogeneous model is no longer rejected or a stopping point is reached. The stopping point is user‐defined, by indicating a minimum number of sequences to be retained for each lineage of interest. If this stopping point is hit, the entire gene is discarded. The approach is applied for each gene or partition independently. LS^4^ essentially extended the approach to also assess extremely slow‐evolving sequences, counterbalancing the very stringent sequence sub‐selection in the presence of such sequences favoured by LS^3^ (Rivera‐Rivera & Montoya‐Burgos, 2019). Hence, in contrast to LS^3^, LS^4^ also removes both slowly and quickly evolving sequences. Although the approach discards entire genes, much like TreeShrink, it does not provide gene‐specific values. Moreover, in this approach, the outgroup is not assessed with respect to branch length heterogeneity. However, outgroup taxa can contribute substantially to the problem of long‐branch attraction (Philippe et al., 2007; Philippe, Brinkmann, Copley, et al., 2011; Pisani et al., 2015; Whelan et al., 2015).

Finally, AliGROOVE is an alignment‐based approach (Kück et al., 2014). The principle of this approach is that sequences affected by increased evolutionary rates—and accordingly long branches—will show more randomly distributed similarity when compared to the other sequences in the dataset. All possible pairs of sequences are compared with each other using a sliding window procedure, which shifts by one position. Within each window, the similarity between the two sequences is determined and compared to similarity scores obtained from 100 Monte Carlo‐resampled windows from a window 3× the size of the sliding window. When the observed score is not significantly different from the random ones each position of the window is assigned either a match or a mismatch. For each position, the matches and mismatches are summed up and normalized by the sliding window size. Finally, for each sequence pair the average across the positions is calculated, resulting in the final similarity score ranging from −1 to 1. These pairwise scores are then displayed in a similarity matrix and can also be plotted on a provided tree. The method can be computationally intensive, as all possible pairs must be calculated, and their number grows exponentially with the number of taxa. Moreover, it can also not be applied to concatenated datasets, as the sliding window is not able to recognize gene boundaries. However, as an alignment‐based approach, tree reconstructions are not necessary, saving computational hours.

### 4.2 Compositional heterogeneity

Compositional heterogeneity comprises, generally spoken, violations of the SRH (stationarity, reversibility, homogeneity) assumptions of substitution models (Jermiin et al., 2016). As a violation of the stationarity assumption also applies to branch length heterogeneity, both heterogeneities are often thought to address the same problem, but this is not always true. Long‐branched sequences can have a homogeneous base composition, while branches of average length can nonetheless exhibit clear signs of base composition heterogeneity (Feuda et al., 2017; Kocot et al., 2016; Nesnidal et al., 2013; Struck et al., 2014).

The impact of SRH violations on topologies can be extreme. A notably extensive study (Naser‐Khdour et al., 2019) compiled 3572 partitions from 35 previously published datasets, and tested which partitions obeyed these three key assumptions using a new metric called the ‘SRH test’ (Jermiin et al., 2016; Naser‐Khdour et al., 2019). This approach tests matched pairs of partitions across an entire dataset, allowing mapping of the total homogeneity across the dataset relative to each partition. To do so, the SRH test requires a topology to compare against. Moreover, for each partition, the two most divergent sequences to one another are used to assess whether they pass or fail a matched‐pair test. Of the partitions, 23.5% violated one or more of these assumptions. In addition, when partitions that violated SRH tests were removed, significantly different tree topologies were recovered (Naser‐Khdour et al., 2019). The SRH test is now included as a standard option in IQ‐Tree to test for violations of these model assumptions (Nguyen et al., 2015).

As a result, one can easily exclude the partitions violating these assumptions (Naser‐Khdour et al., 2019; Nguyen et al., 2015). Unfortunately, due to the comparison of only the two most divergent sequences, the SRH test presents a high risk of both false positives and negative results. A single outlying sequence might fail an entire partition that is not necessarily a strong violation of the model's assumptions. Hence, a dataset is rejected as not being homogeneous even though it might be considered homogeneous when considered as a whole. On the contrary, even though the pair of the two most divergent sequences may not show signs of compositional heterogeneity, it is not necessarily true of the rest of the sequences within the same partition.

In a Bayesian analysis, posterior predictive tests, such as those employed by Phylobayes (Lartillot et al., 2007, 2009), are a reliable way of ascertaining the presence of compositional heterogeneity across a dataset, gene or partition (Feuda et al., 2017). Unfortunately, for each dataset, gene or partition to be investigated, these tests require the output of a Bayesian phylogenetic analysis to obtain the necessary tree lists, and so this analysis can be time‐consuming. Posterior predictive tests simulate replicates of the original data, using the MCMC chain generated during the tree search in Phylobayes to establish reasonable parameters for these new simulated data. By comparing the replicates to the original dataset, values such as the mean number of distinct amino acids in each column, and the variation in observed frequencies across sites can then be compared. This allows us to determine whether the simulated replicates produced by simulating the posterior truly resemble the original dataset. By deriving this new simulated data based on the original, we can quantify whether the original data is significantly different from the expectation, and as such whether the model was a good fit. In case of significant differences, genes, partitions and taxa can be excluded from future analyses. Some studies have been undertaken to evaluate the power of these Bayesian posterior predictive tests in distinguishing systematic errors (Kapli et al., 2021). Taking the currently controversial question of deuterostome monophyly, Kapli et al. originally used Phylobayes' in‐built posterior cross‐validation tests to determine the model that best fits their dataset. Then, Kapli et al. went beyond these options and generated simulation datasets and parameters based on the posterior of their analyses, allowing them to establish which datasets were more likely to be affected by systematic error under which parameters. Though only appropriate for particularly controversial phylogenetic questions, as this kind of simulation analysis is incredibly labour‐intensive, these detailed examinations of the posterior may become more common in the future.

Identification of compositional heterogeneity prior to the analysis can be difficult. The chi‐squared (*χ* ^2^) test for compositional heterogeneity was the first such proposed approach (Cummings, 2004). It is commonly employed and implemented in several programs, including (in a variant form) IQ‐Tree (Nguyen et al., 2015). The *χ* ^2^ test is conducted on the sum of the relative squared difference between the observed occurrences of each base of each sequence to the expected occurrences given the average. However, it suffers in the presence of poorly described data (Foster, 2004; Kumar & Gadagkar, 2001). Compositional heterogeneity can often occur within only a subset of the tree, which a test across an entire dataset may not adequately detect. However, recent implementations, such as those in IQ‐Tree, also allow for the testing of individual sequences by not summing up all sequences in the dataset (Nguyen et al., 2015).

Another tree‐independent method is to calculate normalized relative composition frequency variability (nRCFV) scores as implemented in RCFV Reader (Fleming & Struck, 2022). The nRCFV is the average variability in composition frequency across taxa for a dataset. In addition to the entire dataset, it can also be calculated for subsets of taxa or character states (e.g. purines or pyrimidines, hydrophobic or hydrophilic amino acids), or individual taxa and characters (e.g. only adenine, only leucine). The advantage of nRCFV is that it can be calculated very quickly solely based on the alignment, but it is not related to the model fit or how strongly the assumptions of the model are violated. It also does not by itself provide a statistical test of results. However, tests like student's *t*‐test or outlier tests can be applied to the obtained results. It can, nonetheless, be a valuable tool in exploring data for compositional heterogeneity. For example, observing which parts of a larger dataset are especially strongly affected by compositional heterogeneity and ought to be excluded or included, or determining which taxa could be ‘sequences of interest’ for future analysis and observation (Fleming et al., 2020). This metric was previously implemented in BaCoCa (Kück & Struck, 2014) as RCFV, but was found to be biased towards changes in sequence length and taxa number (Fleming & Struck, 2022). nRCFV introduces a new normalizing constant that ameliorates this issue.

Finally, although the model selection is dealt with extensively in one of the reviews mentioned in our introduction (Kapli et al., 2021; Figure 1), it is worth briefly mentioning again here that if violations of the SRH assumptions occur, one can choose to use more complex mixture models or Lie‐Markov models such as CAT‐GTR or the C‐series (C10, C20, C40, C60), which are more capable of dealing with such violations (Blanquart & Lartillot, 2008; Hannaford et al., 2020; Lartillot et al., 2007). In addition, there are models such as NDCH (Node‐discrete composition heterogeneity), which fits user‐specified areas of the tree to account for localized variation, which is implemented in P4 (Foster, 2004; Foster et al., 2009, 2022; Morgan et al., 2013). Mixture models especially are both growing in their use and appear to be robust to overfitting despite their many parameters (Baños et al., 2022). These are conveniently implemented in many commonly used phylogenetic software, such as CAT‐GTR in Phylobayes (Lartillot et al., 2009) and the C‐series of models in IQ‐Tree (Minh et al., 2020), and so despite their computationally intensive nature, they may prove to be the next step in phylogenetic modelling.

### 4.3 Site saturation

Originally, saturation was detected by plotting uncorrected genetic distances against corrected ones and comparing when the curve began to level to a more consistent value (Xia & Lemey, 2009). From this behaviour, different measurements were developed to allow for direct comparisons of partitions. As such, these early measurements did not allow us to detect taxa affected by site saturation. To overcome this limitation, the linear regression of the pairwise patristic distances against the pairwise uncorrected distance was implemented (Nosenko et al., 2013). Patristic distances are distances based on the branch length in the tree. The lower the slope and *R* ^2^ value of the regression, the more saturated the data are, as unsaturated data should have a slope and *R* ^2^ of 1. This method is now implemented in TreSpEx (Struck, 2014).

The C (convergence) value is a similar, tree‐independent procedure for use on nucleotide data (Struck et al., 2008). The C‐value is based on the ratio of the standard deviation of all transition to transversion ratios and the standard deviation of the uncorrected genetic p distances. With increasing saturation, the standard deviation of the transition to transversion ratio decreases, while the ratio of uncorrected genetic p distances increases. Accordingly, lower C values indicate higher degrees of saturation. The measurement has been implemented in BaCoCa (Kück & Struck, 2014). Both approaches have the disadvantage that they do not provide statistical tests to assess the significance of the values that they produce. However, they are quick to calculate and allow for direct comparison across datasets.

Recently, a change to the entropy‐based measurement for nucleotides (Xia et al., 2003), which is commonly used, has been proposed (Duchêne et al., 2021). The general principle of the entropy‐based measurement for nucleotides is that with increasing entropy, the information content in the dataset decreases. This means that with increasing saturation, less ordered and historic information is in the data, and instead, noise has become predominant. From this, an index for saturation is derived, which is the ratio of the observed information entropy value to a maximum possible value (Xia et al., 2003). This index is compared to a critical index of saturation. If the obtained value is significantly smaller than the critical value, the dataset is not severely affected by saturation. In this recently suggested change, it is additionally tested whether the observed value is significantly smaller than the maximum value, given the critical value (Duchêne et al., 2021). In this way, the variance in the information content across sites is more explicitly considered. As this critical value depends on the topology of the tree, number of taxa, number of sequence positions, assumed distribution of nucleotide frequencies and the substitution rates, it must be determined by simulation analyses. Hence, it is computationally demanding for phylogenomic datasets, as it requires an initial tree topology for the simulations to begin. Alternative procedures use two generic trees for the simulations, one fully symmetrical and one fully asymmetrical. However, this negatively affects the accuracy of the test, as the recovered topology is most likely different from these two extremes.

Recoding strategies might be especially effective in ameliorating artefacts that are caused by base composition heterogeneity or site saturation. One example is the use of 6‐state Dayhoff recoding (Foster et al., 2009), in which an alignment is recoded to best represent the physical properties of the amino acids within the alignment. This assumes that, though there may be a great diversity of sites within the alignment, these will be relatively functionally conserved as the site still corresponds to a similar functional purpose. 6‐state Dayhoff recoding is the most used of these recoding tables (Feuda et al., 2017; Foster et al., 2009, 2022; Hernandez & Ryan, 2019; Marlétaz et al., 2019), compressing the 20‐state amino acid alphabet into 6, but 2‐state nucleotide recoding is also used. 2‐state nucleotide recoding regards only transversions, changes between purines and pyrimidines, to be relevant (Smith et al., 2009). However, recent studies suggest that 6‐state recoding may oversimplify the relationships between amino acids, and that 6‐state recoding may well produce situations that are positively misleading: presenting new, but suspect, results (Hernandez & Ryan, 2019). In this respect, it may be worthwhile to explore more detailed recoding models such as 9, 12, 15, and 18‐state recodings, which do not compress the dataset to such a high degree, in the future (Hernandez & Ryan, 2019). Further work using a combination of both CAT models and Dayhoff recoding (Giacomelli et al., 2022) has suggested that, in the case of models that do not fit the amino acid data well, Dayhoff recoding may improve the fit of the data to the model and, hence, improve accuracy. This suggests that there is an interaction parameter between the model and the recoding strategy that should be considered in future analyses and, as seen in the study itself (Giacomelli et al., 2022), under sufficiently complex models, recoding may not improve the model fit at all. Furthermore, while they did find that their recoded datasets were less saturated when compared to the unrecoded datasets, they also found that the recoded simulations datasets generated under different models did not show significantly different levels of saturation. This suggests that, while Dayhoff recoding reduces site saturation, reducing site saturation may not actually be the mechanism by which it improves model fit and accuracy in phylogenetic datasets (Giacomelli et al., 2022).

Outside of using Dayhoff categories, recoding based on *χ* ^2^ values has also been proposed (Susko & Roger, 2007). In theory, this kind of targeted recoding should reduce base composition heterogeneity, but simulation studies suggest that it may actually decrease accuracy in topology reconstruction in comparison to Dayhoff recoding (Foster et al., 2022). This suggests that merely reducing base composition heterogeneity may not be enough to address site saturation.

Another posterior predictive test to detect site saturation is the relatively simple mean saturation index test (Si Quang et al., 2008). This posterior predictive test quickly generates the most parsimonious tree and compares it to the originally recovered tree to assess differences between the results and the minimum number of reversions per site. Taxa that exhibit site saturation are likely to possess a very high minimum number of reversions per site, as parsimony will be unable to recover their position reliably. Regrettably, this posterior predictive test also requires a phylogenetic result before the hypothesis can be tested, which can be computationally costly.

## 5 CONCLUSIONS

1. Phylogenetic artefacts will be a perennial problem, and even as our models improve, our computers become more powerful and our data becomes comprehensive, evolution is such a complex process that we will continue chasing an approximation of reality.
2. As phylogenies become more important to many forms of broad‐scale systematic work, understanding and evaluating the methods employed will become a crucial skill for interdisciplinary scientists. In this respect, the techniques used to counter these artefacts, and the new methods being developed to aid our understanding of natural history, are not only incredibly exciting, but also fundamental to a broad audience beyond phylogeneticists.
3. With an ever‐increasing amount of data, interfacing and engaging with many of these methods can be difficult, particularly those that take place post hoc, or that require specific technical knowledge to effectively use.
4. Simultaneously, an overly liberal application of many methods of data amelioration risks overfitting data to the model, producing the results we would like to see, rather than the results that the data indicates are there. From a broader viewpoint, we must remember that the bifurcating tree itself is part of this model—a model that assumes that horizontal gene transfer, hybridization and incomplete lineage sorting are negligible.
5. Widespread awareness and use of these tools is not enough—it must be paired with an understanding of why and how these methods work, and where they are and are not suitable.
6. Distinguishing the causes of confounding signals and biological incongruence, and considering which procedures are best in predicting the model violations and the impact they might have on phylogenetic reconstructions requires careful attention, especially when assessing the impact of the hundreds of subsets of taxa and positions that are now becoming available to us.

## CONFLICTS OF INTEREST STATEMENT

The authors have no conflicts of interest.

## FUNDING INFORMATION

This work was supported by the Research Council of Norway Project Number 300587.

## Supporting information


Data S1.

## Data Availability

Not applicable.
