# Supplementary material for: Identifying and addressing methodological incongruence in phylogenomics: A review
Source: Evol Appl. 2023 Jun 6;16(6):1087–104. doi: 10.1111/eva.13565 (PMC10286231; doi:10.1111/eva.13565)
Supplement: Supplementary file 1 — Data S1. [file EVA-16-1087-s001.docx]

# Glossary

**Accuracy:** The likelihood of the hypothesis of the tree representing the relationships between the study sequences in the dataset, given the data.

**Barcoding marker:** A sequence, such as Cytochrome Oxidase I, that is commonly used for taxonomic identification.

**Base composition heterogeneity:** When the proportions of bases in sequences in a dataset are highly variable to one another.

**Bipartition:** A split in the tree that places some sequences on one side, and some on another.

**Bootstrapping:** The creation of pseudo-replicates (see below) by multiplying random positions in a dataset and removing others, to determine the robusticity of a phylogenetic result.

**Branch length heterogeneity:** When the branches in a phylogenetic tree are highly variable when compared to one another. A cause of Long Branch Attraction (see below).

**Bremer Support:** The difference between the most parsimonious tree containing a specific group of taxa as monophyletic (see below), and the most parsimonious tree, where the same taxa do not form a monophyletic group. A high Bremer Support can indicate a high support for a group.

**Coalescence:** The concept that divergent sequences share a common ancestor that shares traits with its descendants, and that the identity of this ancestral sequences can thereby be established for phylogenetic inference purposes. As an allele may be variable in the ancestral population and only fixed in certain descendant lineages, the history of this gene may not be the same as the history of the organism.

**Coverage:** A measure of genome or transcriptome “depth” or quality. The number of unique reads that include a given nucleotide. Higher coverage is better.

**Cross-validation test:** A Bayesian test that compares samples from the posterior (the trees sampled by the Bayesian chain) under different conditions, such as different models, to establish which condition is more likely.

**E-value:** The number of “similarity hits” (significant matches) a BLAST search expects to see by chance when searching a database of that size. A smaller number indicates a more significant hit.

**Entropy:** Also known as “Shannon Entropy” (as opposed to Thermodynamic Entropy). The “information” carried by a dataset or data point, defined as how unlikely it is to occur given the dataset and the model’s assumptions about the data.

**Felsenstein Zone:** The probabilistic region in which phylogenetic inference becomes inconsistent, caused by branch length heterogeneity.

**Gene boundary:** In a concatenated, multi-gene dataset, the transition between two genes.

**Gene conservation:** A measure of the evolutionary substitution rate of particular genes. Highly conserved genes evolve more slowly than ones that are not conserved.

**Homogeneity:** The proportion of character states across a dataset is consistent throughout the dataset. This is an assumption of many phylogenetic models.

**Horizontal gene transfer:** The movement of genetic information between organisms other than from parent to offspring.

**Hybridization:** The process by which an offspring of two distinct species is formed.

**Incomplete lineage sorting:** When an allele has not coalesced prior to a speciation event, resulting in a gene history that is distinct from the species history.

**Incongruence:** Disagreement between gene trees, by methodological or biological causes.

**Information content:** A measure of how useful a parameter or variable is given the dataset. This means information content is relative - as more data is added, or if some is taken away, a variable might become more or less informative, as this produces a different picture of parameter changes (such as changes in character states) across the dataset.

**Information criteria:** The parameters used to establish the information content of a phylogenetic variable (a sequence, taxa, partition, or site).

**Leaf stability:** The stability of a particular bipartition in a tree or set of bipartitions in a tree. A low leaf stability index indicates a tip that is highly prone to movement. Often synonymous with Triplet stability (see below), as when a single bipartition is tested, the tree is simplified to a triplet tree. This is not, however, always true, and the Leaf stability index of a quartet, quintet or larger set could also be tested.

**Lie-Markov model:** A non-reversible phylogenetic model.

**Long branch attraction:** Caused by branch length heterogeneity. A long-branched sequence possesses a longer branch relative to the rest of the dataset, caused by either an increase in rate of change, or a lack of close relatives in the dataset. The degree of change can cause it to artefactually resemble a sequence it is not as closely related to, and thereby gravitate towards an erroneous position in the tree.

**Mean saturation index:** A measure of the average amount of site saturation (see below) in a dataset, and how much the test sequence or partition varies from the average.

**Mixture model:** A model that assigns different substitution rates to different sites in a dataset to account for site saturation (see below).

**Model violation:** When a dataset possesses properties that are not adequately accounted for by the model. Many phylogenetic models assume homogeneity (see above), stationarity, (see below) and reversibility (see below).

**Monophyletic group:** A group of taxa comprising exclusively the descendants of the last common ancestor of these taxa.

**Nodal support:** A family of measures; including posterior probability, Bremer support and Bootstrap support that measure the robusticity or reliability of bipartitions in a dataset.

**Noise:** Data without signal (see below). Data that is too variable, random or misleading to be informative to the model used.

**Ortholog:** Genes in different species that evolved from a common ancestor by a speciation event.

**Outgroup:** A group of sequences that are known to be closely related to the test dataset, but are not part of them, and so form a useful position to root a phylogeny.

**Overfitting:** Adding more parameters than necessary to a model, causing the resulting phylogeny to be more informed by the model than the dataset.

**Paralog:** A gene diverged from another due to a genetic duplication event.

**Parsimonious:** The smallest number of changes necessary.

**Partitioning:** The act of separating a multi-gene dataset into groups with similar biological properties, then applying different models to each group (a partition).

**Patristic distance:** The number of changes separating two taxa along the branches of a tree.

**Polytomy:** A representation of uncertainty in a phylogenetic tree, where rather than bifurcating, a node splits into three or more branches.

**Posterior predictive test:** A form of Bayesian test where data is sampled from the Posterior distribution, and then simulated datasets are generated from these samples, to determine how different the source dataset is from the simulations, and thereby how well fitted the model is to the data.

**Posterior Probabilities:** In Bayesian phylogenetics, often applied as a measure of nodal support. The probability that a bipartition is accurate given the dataset and the model including the prior probabilities.

**Problematic sequences:** A sequence that cannot be accommodated given the current model and dataset.

**Pseudoreplicates:** In bootstrapping, a dataset created by randomly sampling sites from the source dataset to create a new dataset the size of the original, thereby amplifying the signal of certain sites, and masking the signal of others. This measures robusticity of a phylogenetic hypothesis. Compare with Replicate (below).

**Recoding:** Compressing amino acid (20 state) or nucleotide (4 state) data to a smaller number of states, often based on the physical properties of amino acids or nucleotides, to ameliorate the effects of site saturation and base composition heterogeneity.

**Replicate:** A simulation dataset that approximates the properties of the source dataset. Compare with Pseudoreplicate (above).

**Reversibility:** An assumption made by many phylogenetic models that the process of nucleotide and amino acid change is undirected (i.e., the probability of one nucleotide or amino acid exchanging to another is the same in both directions).

**Reversions:** A change from one character state to another, and then back to the original character state.

**Robusticity:** A measure of susceptibility to outliers. Measured by bootstrapping.

**Rogue sequence:** A sequence which cannot be robustly placed anywhere within a phylogenetic topology due to either ambiguous or insufficient phylogenetic information in the dataset.

**Signal:** As opposed to noise (see above). Informative data in a dataset. The signal may not necessarily lead towards a species tree hypothesis, due to incomplete lineage sorting (see above) and other biological causes of incongruence.

**Site saturation:** When particular sites within a dataset are prone to change more frequently than assumed by the model.

**Sliding window procedure:** When a dataset is subsampled a few sites at a time, moving along a dataset one by one, such that each subsample contains elements of both the prior and subsequent “window” subsample.

**Stationarity:** The rates of change between nucleotides or amino acids are the same over the length of the tree.

**Tip-to-root:** The distance from a taxa or sequence (the tip) to the root of the phylogenetic tree.

**Tree diameter:** The distance between the two most unrelated taxa in a dataset.

**Triplet stability:** The stability of a particular bipartition in a tree. A low leaf stability index indicates a tip that is highly prone to movement. Often synonymous with the Leaf Stability index.

**Well-resolved:** A tree with few polytomies.
